# Development and Validation of a Machine Learning Approach for Automated Severity Assessment of COVID-19 Based on Clinical and Imaging Data: Retrospective Study

**DOI:** 10.2196/24572

**Published:** 2021-02-11

**Authors:** Juan Carlos Quiroz, You-Zhen Feng, Zhong-Yuan Cheng, Dana Rezazadegan, Ping-Kang Chen, Qi-Ting Lin, Long Qian, Xiao-Fang Liu, Shlomo Berkovsky, Enrico Coiera, Lei Song, Xiaoming Qiu, Sidong Liu, Xiang-Ran Cai

**Affiliations:** 1 Centre for Health Informatics, Australian Institute of Health Innovation Faculty of Medicine, Health and Human Sciences Macquarie University Macquarie Park Australia; 2 Centre for Big Data Research in Health University of New South Wales Sydney Australia; 3 Medical Imaging Centre The First Affiliated Hospital of Jinan University Guangzhou China; 4 Department of Computer Science and Software Engineering Swinburne University of Technology Melbourne Australia; 5 Department of Biomedical Engineering Peking University Beijing China; 6 Institute of Robotics and Automatic Information System, College of Artificial Intelligence Nankai University Tianjin China; 7 School of Data and Computer Science Sun Yat-sen University Guangzhou China; 8 Department of Radiology Xiangyang Central Hospital Affiliated Hospital of Hubei University of Arts and Science Xiangyang China; 9 Department of Radiology, Huangshi Central Hospital Affiliated Hospital of Hubei Polytechnic University Edong Healthcare Group Huangshi China

**Keywords:** algorithm, clinical data, clinical features, COVID-19, CT scans, development, imaging, imbalanced data, machine learning, oversampling, severity assessment, validation

## Abstract

**Background:**

COVID-19 has overwhelmed health systems worldwide. It is important to identify severe cases as early as possible, such that resources can be mobilized and treatment can be escalated.

**Objective:**

This study aims to develop a machine learning approach for automated severity assessment of COVID-19 based on clinical and imaging data.

**Methods:**

Clinical data—including demographics, signs, symptoms, comorbidities, and blood test results—and chest computed tomography scans of 346 patients from 2 hospitals in the Hubei Province, China, were used to develop machine learning models for automated severity assessment in diagnosed COVID-19 cases. We compared the predictive power of the clinical and imaging data from multiple machine learning models and further explored the use of four oversampling methods to address the imbalanced classification issue. Features with the highest predictive power were identified using the Shapley Additive Explanations framework.

**Results:**

Imaging features had the strongest impact on the model output, while a combination of clinical and imaging features yielded the best performance overall. The identified predictive features were consistent with those reported previously. Although oversampling yielded mixed results, it achieved the best model performance in our study. Logistic regression models differentiating between mild and severe cases achieved the best performance for clinical features (area under the curve [AUC] 0.848; sensitivity 0.455; specificity 0.906), imaging features (AUC 0.926; sensitivity 0.818; specificity 0.901), and a combination of clinical and imaging features (AUC 0.950; sensitivity 0.764; specificity 0.919). The synthetic minority oversampling method further improved the performance of the model using combined features (AUC 0.960; sensitivity 0.845; specificity 0.929).

**Conclusions:**

Clinical and imaging features can be used for automated severity assessment of COVID-19 and can potentially help triage patients with COVID-19 and prioritize care delivery to those at a higher risk of severe disease.

## Introduction

COVID-19 has overwhelmed health systems worldwide [1,2]. Considering the various complications associated with COVID-19 [3-5], methods that help triage patients with COVID-19 can help prioritize care delivery to individuals at a high risk of severe or critical illness. COVID-19 severity can be categorized as follows: mild, ordinary, severe, and critical [6]. Severe and critical cases require intensive care and more health care resources than mild and ordinary cases. A high rate of false-positive severe or critical cases could overwhelm health care resources (ie, beds in the intensive care unit). Moreover, delays in identifying severe or critical cases would lead to delayed treatment of patients at a higher risk of mortality. Therefore, it is important to identify severe cases as early as possible, such that resources can be mobilized and treatment can be escalated.

Chest computed tomography (CT) scans provide important diagnostic and prognostic information [7,8]; consequently, they have been the focus of numerous recent studies using machine learning techniques for COVID-19–related prediction tasks [9-21]. Previous studies have focused on mortality predictions [9], diagnosis (identifying COVID-19 cases and differentiating them from other pulmonary diseases or no disease) [10-15,19,22-25], and severity assessment and disease progression [16-18,23]. Most current approaches have used deep learning methods and imaging features from CT scans [10-15,19,22-24] and X-ray imaging [18,20,21] with popular architectures including ResNet [10,12,14,23], U-Net [11,17], Inception [15,22], Darknet [20], and other convolutional neural networks (NNs) [18,21,26,27]. Recent reviews provide more details regarding these architectures [1,28-32].

Although automated assessment of chest CT scans to predict COVID-19 severity is of great clinical importance, few studies have focused on it [16-18,23]. Automated assessment of chest CT scans can substantially reduce the image reading time for radiologists, provide quantitative data that can be compared across patients and time points, and can be clinically applicable in disease detection and diagnosis, progression tracking, and prognosis [8]. While CT scans are an important diagnostic tool, previous studies reported that clinical data, such as symptoms, comorbidities, and laboratory findings, differed between patients with COVID-19 admitted to intensive care units and those who were not [33], and these data help predict the mortality risk [9]. A previous study compared the imaging data and clinical data of 81 patients with confirmed COVID-19 and suggested that the combination of imaging features with clinical and laboratory findings facilitated an early diagnosis of COVID-19 [34].

In this study, we used patient clinical data and imaging data to predict disease severity among patients with COVID-19. Considering this as a putative binary classification task, we predicted whether a patient diagnosed with COVID-19 is likely to have mild or severe disease. This study has 3 objectives. First, we compared the predictive power of clinical and imaging data for disease severity assessment by testing three machine learning models: logistic regression (LR) [35], gradient boosted trees (eg, XGBoost) [36], and NNs [37]. Second, since our cohort data are highly imbalanced, with the majority of cases being of mild/ordinary severity, we tested 4 oversampling methods to address the imbalanced classification issue [38-41]. Third, we interpreted the importance of features by using the Shapley Additive Explanations (SHAP) framework and identified features with the highest predictive power [42]. The predictive models evaluated herein yielded high accuracy and identified predictive imaging and clinical features consistent with those reported previously.

## Methods

### Participants

This retrospective study was performed using data collected by 2 hospitals in the Hubei Province, China. The study cohort consisted of patients with COVID-19 diagnosed through RT–PCR analysis of nasopharyngeal swab samples. A total of 346 patients from 2 hospitals were retrospectively enrolled, including 230 (66.5%) patients from Huang Shi Central Hospital (HSCH) and 116 (33.5%) from Xiang Yang Central Hospital (XYCH). These patients were admitted to hospital between January 1 and February 23, 2020, and underwent chest CT upon initial hospitalization. All participants provided written consent. This study was approved by the institutional review board of both hospitals (approval number LL-2020-032-02). Table 1 summarizes the demographic characteristics of the patients in the 2 cohorts.

**Table 1:** Demographic characteristics of the patients in the 2 cohorts (N=346).

Category		Variables		
		HSCH^a^ (n=230)	XYCH^b^ (n=116)	Total
**COVID-19 severity, n (%)**				
	Mild	7 (3.0)	1 (0.9)	8 (2.3)
	Ordinary	212 (92.2)	104 (89.7)	316 (91.3)
	Severe	7 (3.0)	6 (5.2)	13 (3.8)
	Critical	4 (1.7)	5 (4.3)	9 (2.6)
Age (years), mean (SD)		49.0 (14.4)	47.5 (17.2)	48.5 (15.4)
Gender ratio (female to male)		120:110	57:59	177:169

^a^HSCH: Huang Shi Central Hospital.

^b^XYCH: Xiang Yang Central Hospital.

### Imaging and Clinical Data

**Figure 1:**
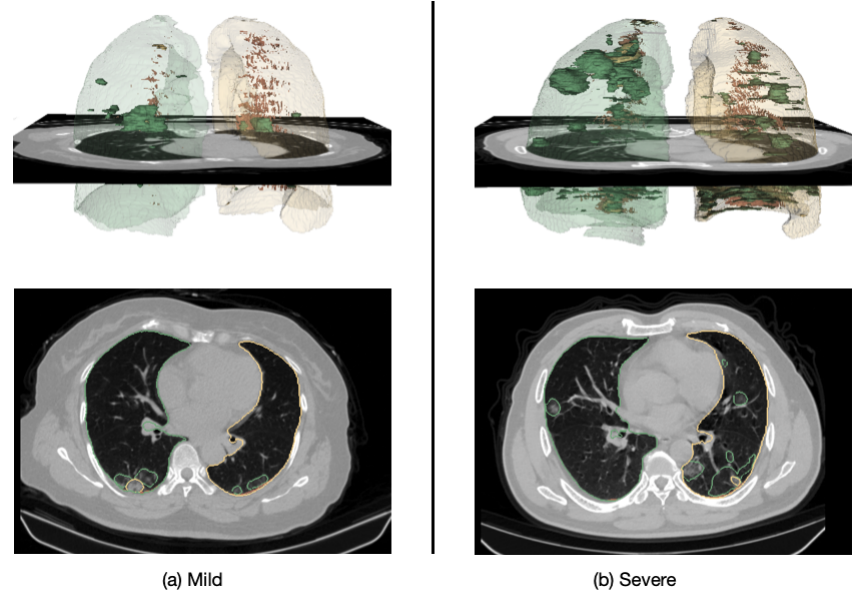
Representative chest computed tomography scans and the lung and lesion models of (A) a mild COVID-19 case and (B) a severe COVID-19 case.

Chest CT scans of patients were collected upon initial hospitalization and preprocessed using intensity normalization, contrast limited adaptive histogram equalization, and gamma adjustment, using the same preprocessing pipeline as in our previous study [43]. We performed lung segmentation in the chest CT images by using an established model “R231CovidWeb” [44], which was pretrained using a large, diverse data set of non–COVID-19 chest CT scans and further fine-tuned with an additional COVID-19 data set [45]. CT slices with <3 mm^2^ of lung tissue were excluded from the data sets since they provide limited or no information about the lung. Lung lesions were segmented using EfficientNetB7 U-Net [16], which was also pretrained using a public COVID-19 data set [45]. The model indicated four types of lesions: ground-glass opacities, consolidations, pleural effusions, and other abnormalities. The volume of each lesion type and the total lesion volume were calculated from the segmentation maps as the imaging features and were further normalized by the lung volume. Figure 1 shows representative results of lung and lesion segmentation of a mild case and a severe case, wherein the upper row presents 3D models of the lung and lesions reconstructed using 3D Slicer (v4.6.2) [46], and the lower row presents axial chest CT slices with the lung and lesion (green: ground-glass opacities, yellow: consolidation, and brown: pleural effusion) boundaries overlaid on the CT slices.

Clinical data collected from the patients included demographic characteristics, signs, symptoms, comorbidities, and the following 18 laboratory findings: white blood cell count (×10^9^/L), neutrophil count (×10^9^/L), lymphocyte count (×10^9^/L), hemoglobin (g/L), platelets (×10^9^/L), prothrombin time (s), activated partial thromboplastin time (s), D-dimer (nmol/L), C-reactive protein (mg/L), albumin (g/L), alanine aminotransferase (µkat/L), aspartate aminotransferase (µkat/L), total bilirubin (µmol/L), potassium (mmol/L), sodium (mmol/L), creatinine (µmol/L), creatine kinase (µkat/L), and lactate dehydrogenase (µkat/L).

**Figure 2:**
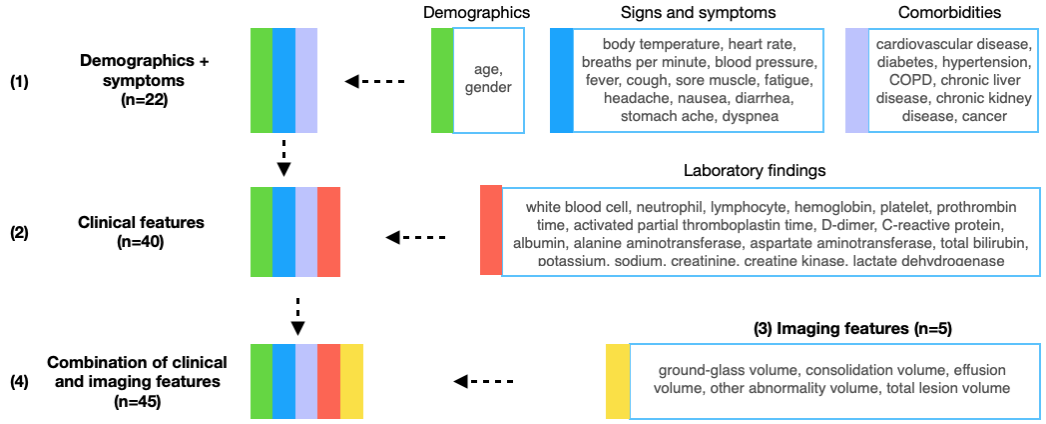
Structure and dimensions of the feature sets. COPD: chronic obstructive pulmonary disorder.

All features were either continuous or binary—all binary features include signs, symptoms, and comorbidities. Continuous features were standardized to be centered around 0 (SD 1). Figure 2 shows the structure and dimensions of the features used in this study. These features were grouped into four feature sets: demographic characteristics and symptoms (a subset of the available clinical features), clinical features (demographic characteristics, signs and symptoms, and laboratory findings), imaging features extracted from the chest CT scans through deep learning methods, and a combination of clinical and imaging features.

### Severity Assessment Models

We trained and compared three models to predict case severity: LR (with scikit-learn) [47], gradient boosted trees (XGBoost) [36], and an NN (fast.ai) [48]. We used the HSCH data (230 samples) for training and validation using 5-fold repeated stratified cross-validation. The XYCH data (116 samples) were withheld for testing. We reported the results for the test set with the area under the curve (AUC) and F1 scores averaged through independent runs.

Hyperparameter exploration and tuning were performed using the training/validation set. A random search was performed to tune the hyperparameters of LR and XGBoost. For NN, we used a 4-layer, fully connected architecture, with the first hidden layer having 200 nodes and a second hidden layer of 100 nodes. We determined the learning rate (0.01) using Learning Rate Finder [49]. All other NN parameters were set to default values. We explored a different number of nodes in the first and second hidden layers, with 200×100 images yielding the best results in the validation set. Of 346 patients, 167 (48%) had at least one missing feature (5.7 on average, mostly for the laboratory findings). Missing feature values were imputed with the mean for each feature.

### Oversampling

The majority of cases in our data set were of mild/ordinary severity, with only a few cases of severe/critical severity. The imbalance ratio for the entire data set was 0.07; training/validation set, 0.05; and testing set, 0.10. We tested four oversampling methods to increase the ratio of the minority class: synthetic minority oversampling (SMOTE) [38], Adaptive Synthetic sampling [39], geometric SMOTE [40], and a conditional generative adversarial network (CTGAN) model for tabular data [41]. For these methods, we oversampled the training set, trained a model using the oversampled data, and reported results on the same test set. We adjusted the resampling ratio of all methods to 0.3 (thus setting the imbalance ratio to 0.3). Using CTGAN for oversampling, we fitted the CTGAN model with the training set, performed sampling to generate synthetic data, using only synthetic data for the minority class (severe/critical), and this was repeated until the minority-to-majority class ratio approached 0.3.

## Results

### Patient Characteristics

Table 2 summarizes the patients’ characteristics. The differences between the mild/ordinary and severe/critical groups were assessed with the Mann-Whitney *U* test and Fisher exact test. The median age of the entire cohort was 49 (IQR 38-59) years. The median age of patients with mild/ordinary COVID-19 was 48.5 (IQR 37.0-57.3) years and that of patients with severe/critical COVID-19 was 63.0 (IQR 52.5-69.5) years. We observed significant differences between patients with severe/critical COVID-19 and those with mild/normal COVID-19 with respect to age (*P*<.001) and comorbidities including cardiovascular disease (*P*=.002), hypertension (*P*=.002), diabetes (*P*=.01), and cancer (*P*=.01). From among all signs and symptoms, an increased respiration rate (*P*=.002) and dyspnea (*P*<.001) were more common among patients with severe/critical COVID-19 than among those with mild/ordinary COVID-19.

**Table 2:** Demographics and baseline characteristics of patients with confirmed COVID-19 (N=346). Symptoms including cardiovascular disease and shortness of breath were more likely in cases of severe/critical COVID-19.

Characteristics				Patients						
				All patients		Mild/ordinary		Severe/critical		*P* value^a^
Sample size, n				346		324		22		N/A^b^
**Demographic characteristics**										
	Age (years), median (IQR)			49.0 (38.0-59.0)		48.5 (37.0-57.3)		63.0 (52.5-69.5)		<.001
	**Gender, n (%)**									.38
		Female	177 (51.2)		168 (51.9)		9 (41.0)			
		Male	169 (48.8)		156 (48.1)		13 (59.0)			
**Comorbidities, n (%)**										
	Cardiovascular disease			40 (11.6)		32 (9.9)		8 (36.0)		.002
	Diabetes			34 (9.8)		28 (8.6)		6 (27.0)		.01
	Hypertension			51 (14.7)		42 (13.0)		9 (41.0)		.002
	Chronic obstructive pulmonary disease			11 (3.2)		9 (2.8)		2 (9.0)		.15
	Chronic liver disease			7 (2.0)		7 (2.2)		0 (0)		N/A
	Chronic kidney disease			4 (1.2)		3 (0.9)		1 (5.0)		.20
	Cancer			8 (2.3)		5 (1.5)		3 (14.0)		.01
**Signs, median (IQR)**										
	Body temperature			37.8 (37-38.3)		37.8 (37-38.3)		38.1 (37.1-39)		.11
	Heart rate			85 (80-90)		85 (80-90)		90 (80-101.8)		.11
	Breaths per minute			20 (20-21)		20 (20-21)		21 (20-28)		.002
	Blood pressure high			120 (119.5-130.0)		120 (118.5-130.0)		127 (120-146.5)		.07
	Blood pressure low			74 (69-80)		74 (69-80)		79.5 (71-89)		.08
**Symptoms, n (%)**										
	Fever			275 (79.5)		256 (79.0)		19 (86.0)		.59
	Cough			238 (68.8)		220 (67.9)		18 (82.0)		.24
	Fatigue			118 (34.1)		108 (33.3)		10 (45.0)		.25
	Dyspnea			32 (9.2)		23 (7.1)		9 (41.0)		<.001
	Sore muscle			38 (11.0)		35 (10.8)		3 (14.0)		.72
	Headache			34 (9.9)		31 (9.6)		3 (14.0)		.47
	Diarrhea			23 (6.6)		20 (6.2)		3 (14.0)		.17
	Nausea			9 (2.6)		7 (2.2)		2 (9.0)		.11

^a^*P* values were compared using mild/ordinary and severe/critical cases were obtained with Mann-Whitney *U* test and Fisher exact test. As no patient in our cohort had a stomach ache, this feature was not factored into our model.

^b^N/A: not applicable.

### Prediction of COVID-19 Severity at Baseline

Data from the HSCH (230 patients, 66.5%) were used for training and validation, and data from the XYCH (116 patients, 33.5%) were used as the independent test set. We compared model performance using four feature sets: demographic characteristics and symptoms, clinical features, imaging features, and a combination of clinical and imaging features (Figure 2). The optimal classification threshold for the sensitivity, specificity, and F1 score was identified using the Youden index [50]. Table 3 shows the severity assessment performance of an LR model, an XGBoost model, and a 4-layer fully connected NN model. Overall, LR models outperformed the other evaluated models, achieving the highest AUC, F1 score, and sensitivity for all four feature sets. While imaging features yielded substantially better results than clinical features, the combination of clinical and imaging features benefited only the LR model. Hence, the LR model displayed the best performance (AUC 0.950; F1 score 0.604; sensitivity 0.764; specificity 0.919) upon using the combination of clinical and imaging features.

**Table 3:** Results of using different feature sets (values in italics indicate the best results).

Feature sets and model			Area under the curve		F1 score		Sensitivity	Specificity
**Demographics + symptoms**								
	LR^a^	*0.819*		*0.382*		*0.627*		0.825
	XGB^b^	0.763		0.363		0.318		*0.956*
	NN^c^	0.730		0.332		0.427		0.880
**Clinical**								
	LR	*0.848*		*0.387*		*0.455*		0.906
	XGB	0.787		0.286		0.227		*0.962*
	NN	0.647		0.237		0.309		0.881
**Imaging**								
	LR	*0.926*		*0.593*		*0.818*		0.901
	XGB	0.904		0.486		0.636		0.896
	NN	0.845		0.555		0.600		*0.936*
**Clinical + imaging**								
	LR	*0.950*		*0.604*		*0.764*		0.919
	XGB	0.904		0.520		0.473		*0.965*
	NN	0.782		0.413		0.486		0.907

^a^LR: logistic regression.

^b^XGB: XGBoost.

^c^NN: neural network.

### Prediction at Baseline Severity With Oversampling

**Figure 3:**
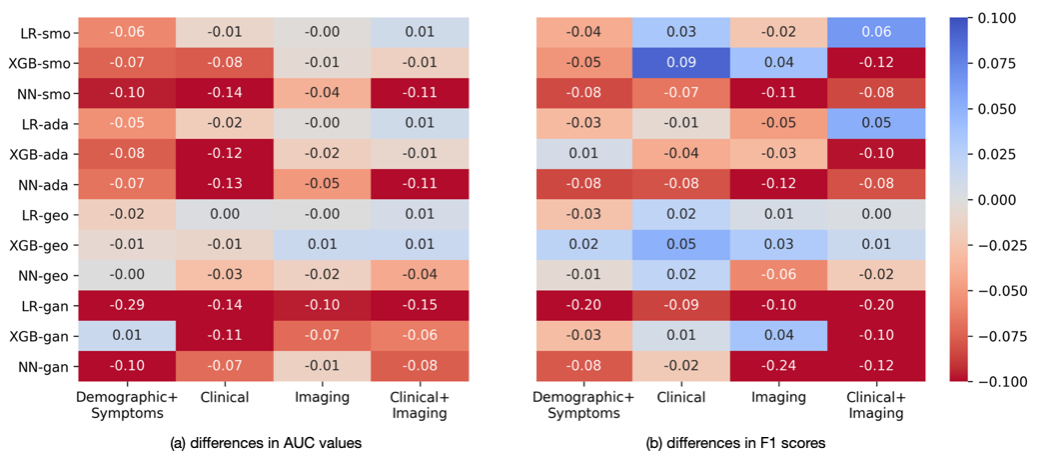
Differences in the (A) area under the curve values and (B) F1 scores with oversampling and without oversampling. Positive values (blue) indicate oversampling resulting in higher values, negative values (red) indicating oversampling resulting in lower values. smo: synthetic minority oversampling; ada: Adaptive Synthetic sampling; geo = geometric synthetic minority oversampling; gan: conditional generative adversarial network; LR: logistic regression; NN: neural network; XGB: XGBoost.

Since the cohort was highly imbalanced, with the majority of cases being of mild/ordinary severity (imbalance ratio 0.07), we applied four oversampling methods to increase the ratio of severe/critical cases: SMOTE [38], Adaptive Synthetic sampling [39], geometric SMOTE [40], and CTGAN [41]. Figure 3 shows the differences in AUC values and F1 scores obtained through oversampling, with negative values indicating a reduction in AUC or F1 scores and positive values indicating the opposite trend. Oversampling resulted in greater improvements in the F1 score than in the AUC. The greatest improvement in the F1 score (0.09) was observed for the clinical features (clinical) with XGBoost and SMOTE (XGB-smo); however, the AUC decreased by 0.08 with the same method. Considering both AUC and F1 scores simultaneously, the combination of clinical and imaging features (clinical + imaging) benefited most from oversampling. In particular, the AUC and F1 score for clinical + imaging features were increased by 0.01 and 0.06, respectively, using LR with SMOTE (LR-smo).

Table 4 presents the best results of the evaluated models using various feature sets after oversampling. Oversampling did not improve the performance of the LR model for the demographic characteristics + symptoms features, but SMOTE and geometric SMOTE increased the F1 scores for clinical features and imaging features, respectively. Notably, the performance of the LR model (Table 3) was optimal for the combination of clinical and imaging features, with improvements in the AUC (0.960 vs 0.950), F1 score (0.668 vs 0.604), sensitivity (0.845 vs 0.764), and specificity (0.929 vs 0.919), after oversampling with SMOTE.

**Table 4:** The best results obtained using different feature sets after oversampling (arrow indicates improved performance after oversampling).

Feature sets	Results				
	Model	Area under the curve	F1	Sensitivity	Specificity
Demographics + symptoms	LR^a,b^	0.819	0.382	0.627	0.825
Clinical	LR – smo^c^	0.837	0.421 ↑	0.518 ↑	0.902
Imaging	LR – geo^d^	0.926	0.599 ↑	0.818	0.904 ↑
Clinical + imaging	LR – smo	0.960 ↑	0.668 ↑	0.845 ↑	0.929 ↑

^a^LR: logistic regression.

^b^No improvement after oversampling.

^c^smo: synthetic minority oversampling.

^d^geo: geometric synthetic minority oversampling.

### Model Interpretation

We used the SHAP framework [42] to interpret the output of the best-performing LR model through SMOTE oversampling. This framework helps determine the importance of a feature by comparing model predictions with or without the feature.

**Figure 4:**
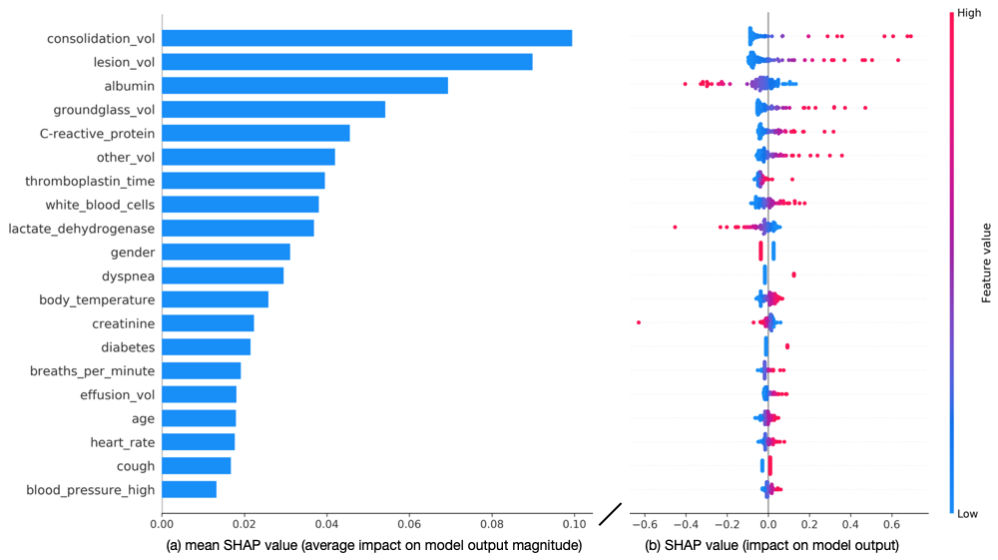
(A) Feature importance, evaluated using the mean SHAP (Shapely Addictive Explanations) values, in the logistic regression (LR) model using all features. (B) SHAP plot for the LR model using all features. Each point represents a feature instance, and the color indicates the feature value (red: high, blue: low). Negative SHA*P* values indicate feature instances contributing to a model output of a mild/ordinary COVID-19 case, whereas positive SHA*P* values indicate features contributing to a model output of a severe/critical COVID-19 case.

Figure 4 shows a SHAP plot summarizing how the values of each feature impact the model output of the LR model using all features (clinical and imaging features), with features sorted in descending order of importance. Figure 4A shows the feature importance scores sorted by the average impact on the model output, and Figure 4B shows the SHA*P* values of individual features. Furthermore, 4 imaging features, including consolidation volume (consolidation_val), total lesion volume (lesion_vol), ground-glass volume (groundglass_vol), and volume of other abnormalities (other_vol), are among the top 6 features, their high values increasing the likelihood of the model to predict a severe/critical COVID-19 case. Low albumin levels, high C-reactive protein levels, a high leukocyte count, and low lactate dehydrogenase levels make the model more likely to predict a critical/severe COVID-19 case. Moreover, older age and male gender increased the likelihood of the model to predict severe/critical COVID-19 cases.

## Discussion

### Principal Findings

In our cohort of patients with COVID-19, fever, cough, and fatigue were the most common symptoms, consistent with previous studies on COVID-19 [34]. The incidence of dyspnea and an increased respiratory rate was significantly higher in severe cases. Some symptoms such as sore muscle, headache, diarrhea, and nausea were present in 9–38 (2.6%-11.0%) of patients and did not differ significantly between mild and severe cases. Patients with severe COVID-19 tended to be of older age and had comorbidities (including cardiovascular disease, diabetes, hypertension, and cancer), concurrent with previous studies [1,3,5,34]. We observed no difference between males and females in our cohort, although the model did rely on gender for increasing the likelihood of predicting a severe/critical case.

A combination of clinical and imaging features yielded the best performance. Imaging features had the strongest impact on model output, with high values of consolidation volume, lesion volume, ground-glass volume, and other volume increasing the likelihood of the model to predict a severe case of COVID-19. Ground-glass opacity is an important feature of COVID-19 [14]. The inclusion of clinical features further improved the accuracy of severity assessment, with findings such as albumin levels, C-reactive protein levels, thromboplastin time, white blood cell counts, and lactate dehydrogenase levels being amongst the most informative features, concurrent with a previous study that also used laboratory findings to predict COVID-19–related mortality [9]. Furthermore, C-reactive protein was associated with a significant risk of critical illness in a study of 5279 patients with laboratory-confirmed COVID-19 [5]. Our model also relied on symptoms and patient characteristics such as gender, dyspnea, body temperature, diabetes, and respiratory rate for differentiating between mild and severe cases. Clinical features alone (demographics, signs, symptoms, and laboratory results), resulted in low sensitivity. Therefore, dependence on only clinical features poses the risk of predicting mild/ordinary COVID-19 among patients at the risk of critical/severe illness.

Oversampling yielded mixed results, although it revealed the best model performance in our study. The best model without oversampling (ie, the LR model) also yielded remarkable findings (AUC 0.950; F1 0.604; sensitivity 0.764; specificity 0.919), and SMOTE oversampling further improved the model performance (AUC 0.960; F1 0.668; sensitivity 0.845; specificity 0.929). Considering the propensity of health care data to be imbalanced [51-54], our results suggest the need for further analysis of oversampling methods for medical data sets. Self-supervision [55,56] may also help improve the performance of models using imbalanced medical data sets; in particular, future studies should evaluate the impact of self-supervision on tabular medical data.

### Clinical Implications

The rapid spread of COVID-19 has overwhelmed health care systems, necessitating methods for efficient disease severity assessment. Our results indicate that clinical and imaging features can facilitate automated severity assessment of COVID-19. While our study would benefit from a larger data set, our results are encouraging because we trained the models with data from one hospital only and tested them using an independent data set from another hospital, albeit with high predictive accuracy.

The proposed methods and models would be useful in several clinical scenarios. First, the proposed models are fully automated and can expedite the assessment process, saving time in reading CT scans or evaluating patients through a scoring system. These models can be useful in hospitals that are overwhelmed by a high volume of patients during the outbreak by identifying severe cases as early as possible, such that treatment can be escalated. Our models, with low sensitivity and high specificity, are best used in combination with a model with high sensitivity and low specificity. A high-sensitivity model can identify patients with severe COVID-19, and our model (with high specificity) could identify false-positives; that is, patients with mild COVID-19 who were wrongly identified as having severe COVID-19.

Our models were developed and validated using 4 different feature sets, providing the flexibility to accommodate patients with different available data. For example, if a patient has neither a chest CT scan nor a blood test, the model based on demographics and symptoms can still achieve reasonably good prediction performance (AUC 0.819; sensitivity 0.627; specificity 0.825). Availability of the patients’ clinical and imaging features can improve the model’s sensitivity and specificity, with the potential to triage patients with COVID-19 (eg, prioritizing care for patients at a higher risk of mortality).

### Limitations and Future Prospects

Our data set consisted of 346 patients with confirmed COVID-19, with data on 230 (66.5%) patients from HSCH used for training/validation and data on 116 (33.5%) patients from XYCH used for testing. Our data set was highly imbalanced, which could have made models overfit to the majority class. In addition, only the baseline data for patients were used in this study; therefore, we could not assess how early can COVID-19 progression be detected. We intend to further investigate the longitudinal data and design computational models to predict disease progression in our future studies.

While we explored various NN configurations, the results were not comparable to those of LR, presumably owing to the limited data set and the low dimensionality of the feature vectors. In this study, we used a complex NN model (EfficientNetB7 U-Net) to extract the imaging features and tested various models for classification using the combination of imaging features and tabular clinical data. Such 2-stage processing may simplify the classification task for these models, thereby reducing the need for another NN model for classification owing to low dimensionality of the features. Further exploration of NN architectures for tabular data would likely improve the performance of the NN model, especially if more data are available.

During training and validation, the performance of the models across cross-validation folds showed high variance owing to the small number of positive cases in the validation fold. A larger dataset would improve the reliability and robustness of the models. The data also consisted of COVID-19 cases which were confirmed through RT–PCR analysis of nasopharyngeal swabs. As such, our model is limited to differentiating severe/critical cases from mild/ordinary cases of COVID-19 and not for diagnosing COVID-19 or differentiating COVID-19 cases from those of other respiratory tract infections. Further studies are required to determine the efficacy of the severity assessments, including data from asymptomatic patients.

Using the Prediction Model Study Risk of Bias Assessment Tool [57], our models are at a high risk of bias owing to a potential bias in the participants domain (the cohort including participants [mean age 48.5 years, SD 15.4 years] who were admitted to hospitals) and the analysis domain (small sample size and class imbalance). Our models are at a low risk of bias in the predictor and outcome domains.

### Conclusions

This study presents a novel method for severity assessment of patients diagnosed with COVID-19. Our results indicate that clinical and imaging features can be used for automated severity assessment of COVID-19. While imaging features had the strongest impact on the model’s performance, inclusion of clinical features and oversampling yielded the best performance in our study. The proposed method may potentially help triage patients with COVID-19 and prioritize care for patients at a higher risk of severe disease.

## Abbreviations

AUC: area under the curve
CT: computed tomography
CTGAN: conditional generative adversarial network
HSCH: Huang Shi Central Hospital
LR: logistic regression
NHMRC: National Health and Medical Research Council
NN: neural network
SHAP: Shapley Additive Explanations
SMOTE: synthetic minority oversampling
XYCH: Xiang Yang Central Hospital
